# Interactions of HIV-1 Capsid with Host Factors and Their Implications for Developing Novel Therapeutics

**DOI:** 10.3390/v13030417

**Published:** 2021-03-05

**Authors:** Shentian Zhuang, Bruce E. Torbett

**Affiliations:** 1 Department of Immunology and Microbiology, The Scripps Research Institute, La Jolla, CA 92037, USA; Shentian.Zhuang@seattlechildrens.org; 2 Center for Immunity and Immunotherapies, Seattle Children’s Research Institute, Seattle, WA 98101, USA; 3 Department of Pediatrics, University of Washington School of Medicine, Seattle, WA 98105, USA

**Keywords:** HIV-1 capsid, host factors, capsid-targeting inhibitors, high-throughput screening techniques, antiretroviral therapeutics

## Abstract

The Human Immunodeficiency Virus type 1 (HIV-1) virion contains a conical shell, termed capsid, encasing the viral RNA genome. After cellular entry of the virion, the capsid is released and ensures the protection and delivery of the HIV-1 genome to the host nucleus for integration. The capsid relies on many virus–host factor interactions which are regulated spatiotemporally throughout the course of infection. In this paper, we will review the current understanding of the highly dynamic HIV-1 capsid–host interplay during the early stages of viral replication, namely intracellular capsid trafficking after viral fusion, nuclear import, uncoating, and integration of the viral genome into host chromatin. Conventional anti-retroviral therapies primarily target HIV-1 enzymes. Insights of capsid structure have resulted in a first-in-class, long-acting capsid-targeting inhibitor, GS-6207 (Lenacapavir). This inhibitor binds at the interface between capsid protein subunits, a site known to bind host factors, interferes with capsid nuclear import, HIV particle assembly, and ordered assembly. Our review will highlight capsid structure, the host factors that interact with capsid, and high-throughput screening techniques, specifically genomic and proteomic approaches, that have been and can be used to identify host factors that interact with capsid. Better structural and mechanistic insights into the capsid–host factor interactions will significantly inform the understanding of HIV-1 pathogenesis and the development of capsid-centric antiretroviral therapeutics.

## 1. Introduction

Human Immunodeficiency Virus type 1 (HIV-1) is a lentivirus containing two RNA genomes that must be reverse transcribed into double-stranded DNA and then integrated into the host genome to ensure a productive infection [1]. HIV-1 can infect host CD4^+^ T cells and macrophages, resulting in CD4^+^ T cell loss and immune dysfunction that leads to the acquired immune deficiency syndrome (AIDS) [2,3].

HIV-1 has evolved to usurp the host machinery to replicate and spread, as well as to counteract host immune defenses. These processes are achieved mainly from the physical interactions between viral and host cellular proteins, which contribute to the uniqueness of HIV-1 infection [4,5].

**Figure 1:**
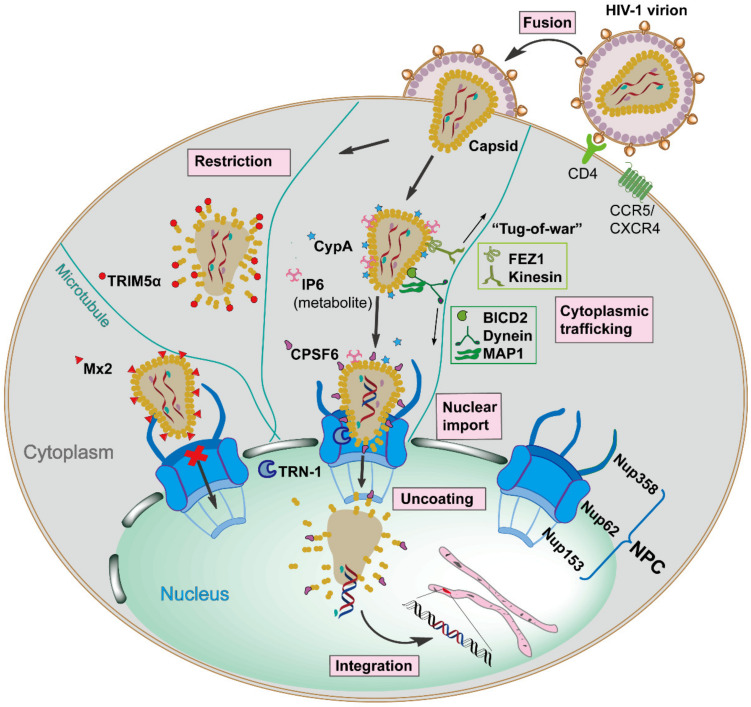
Schematic overview of the early stages of Human Immunodeficiency Virus type 1 (HIV-1) replication, which is spatiotemporally regulated by diverse capsid-interacting host factors. The HIV-1 virion binds to the CD4 receptor and the CXCR4 and/or CCR5 coreceptors via the viral envelope glycoproteins, resulting in fusion with the target cell, which releases capsid into the cellular cytoplasm. The capsid traffics towards the nucleus along the microtubule network by employing opposing adaptor and motor proteins such as FEZ1, kinesin, BICD2, dynein, and MAP1, a process that may result in a bi-directional “tug-of-war”. The host cellular protein CypA and metabolite IP6 bind to capsid and maintain core stability during cytoplasmic trafficking. The host innate immune system can respond to capsid, resulting in interferon (IFN) expression and inducing the IFN-induced viral restriction factors TRIM5α and Mx2. TRIM5α binds to the capsid causing premature uncoating, while Mx2 binds to the capsid impeding the nuclear import. When capsid arrives at the nuclear envelope, it presumably binds Nup358, and other factors, to promote import into the nucleus via the nuclear pore complex as an intact or nearly intact capsid. The host factors CPSF6, Nup153, and TRN-1 also appear to facilitate capsid nuclear import via direct capsid interactions. Reverse transcription is completed in the nucleus, followed by complete uncoating of the capsid and integration of the viral DNA into the host genome. All processes are detailed in the text.

HIV-1 infection relies on the viral envelope to bind to the host CD4 receptor and CXCR4 or CCR5 coreceptors for attachment and fusion, resulting in the release of the viral core, capsid, into the cellular cytoplasm [6]. Capsid is comprised of ~250 hexamers and exactly 12 pentamers, assembled from ~1500 copies of monomeric capsid proteins (monomer is defined as CA herein) [7]. This conical capsid houses the viral RNA genome, and the replicative enzymes reverse transcriptase (RT) and integrase (IN), which are required for reverse transcription and integration [8]. Once capsid is present in the cytoplasm, it traffics along the microtubule network to the nuclear envelope, and then is imported into the nucleus in an intact or nearly intact state via the nuclear pore complex (NPC) [9,10]. Reverse transcription appears to be completed in the nucleus, followed by complete uncoating of the capsid and integration of the viral DNA into the host cell genome [11]. However, the exact mechanisms of capsid nuclear entry and when uncoating starts remain to be determined. These events are the early stages of the HIV-1 replication cycle (see Figure 1 for an overview). The successfully integrated viral genome is then transcribed and translated into viral proteins by host machinery to generate new infectious virions, defined as late stages of the HIV-1 replication cycle [12], which will not be discussed in detail in this review.

Capsid performs a protective role by shielding the viral genome from host innate immune detection and provides a micro-environment for reverse transcription [13,14]. Moreover, the exterior surface of the capsid has functions beyond the simple encapsidation by providing an expansive area for recognition by diverse cellular proteins acting as either pro-viral or anti-viral factors [15,16,17,18]. Noticeably, these host factors specifically bind to unique high-order interfaces only present in the assembled capsid lattice, and they have no or low affinity to CA monomers, underscoring the fact that capsid structural integrity is critical for the interplay with host factors [19]. In the first part of this review, we aim to summarize the well-known physical interactions between capsid and diverse host proteins and their functional roles, which undoubtedly hold the key for driving HIV-1 early infection events.

Antiretroviral therapy (ART) has turned AIDS from a ‘virtual death sentence’ to a ‘chronic manageable disease’ by targeting and inhibiting the function of viral enzymes (reverse transcriptase, integrase, and protease) [20]. However, the emergence of ART resistance, the absence of an effective vaccine, and the need for long-lasting anti-retroviral compounds to enhance treatment adherence, are reasons for continued research to develop new therapeutics [21,22,23]. A number of non-viral enzymatic targets have been investigated as possible antiretroviral targets. One successful example is the development of the co-receptor antagonist maraviroc which blocks the cellular entry of HIV-1 by preventing the interaction between viral envelope and CCR5 [24,25]. This is the first and only Food and Drug Administration (FDA)-approved anti-HIV drug targeting a host cellular protein. Due to the multiple roles capsid plays during infection, it has been exploited as a target for anti-retroviral compound screening [26,27]. A handful of compounds have been reported to bind capsid and disrupt infection, of which, PF-3450074 (PF74) and the Gilead compounds, GS-CA1 and GS-6207, are potent inhibitors [28]. GS-6207 has entered clinical trials [29]. These potent capsid inhibitors have similar scaffolds and share the same capsid binding pocket with the host proteins CPSF6 and Nup153, which both mediate capsid nuclear entry [29]. These findings underscore the value of investigating whether additional host factor-binding sites on capsid can be identified and exploited for novel therapeutic interventions. These topics will be discussed in the second part of this review.

Further to the point of identifying and investigating capsid–host factor binding, which may allow small molecule targeting, HIV–host factor identification studies have benefited significantly from the application of high-throughput “-omic” screening techniques [30,31]. In the last part of this review, we will briefly introduce the genome- and proteome-based approaches that have been and can be used to characterize virus–host interactions in HIV-1 or other viruses and discuss the potential they hold for identifying new capsid-interacting proteins.

## 2. The Dynamic Capsid–Host Interactions during Early HIV-1 Infection

### 2.1. HIV-1 Capsid Architecture

**Figure 2:**
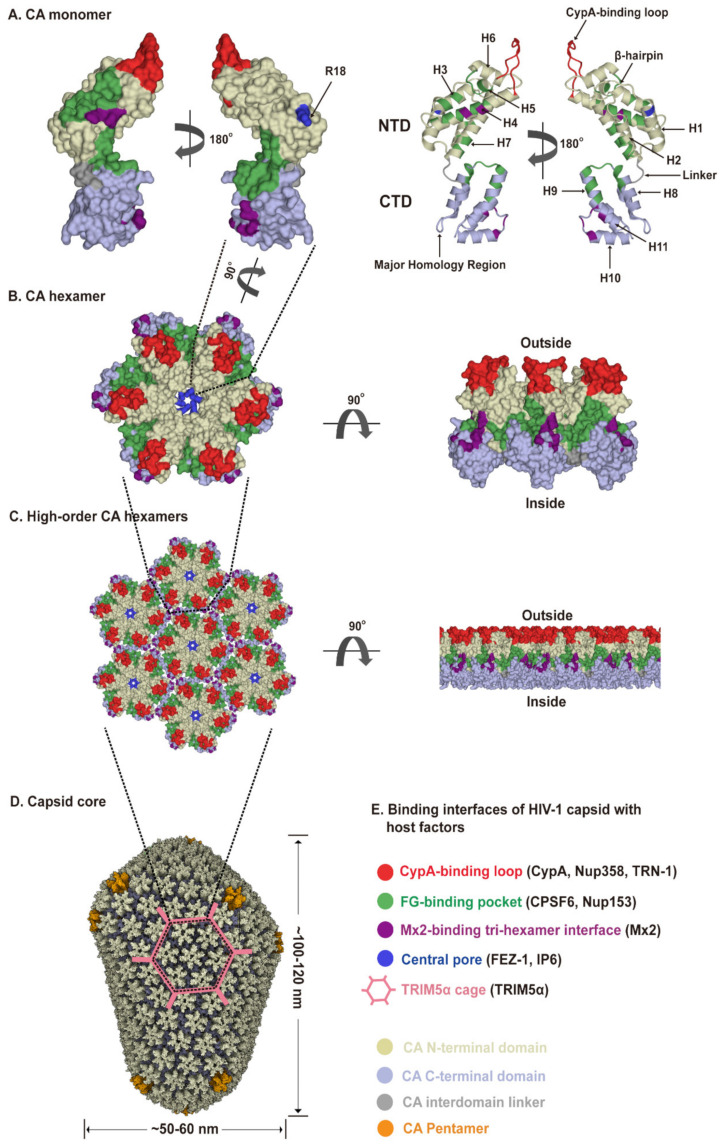
HIV-1 capsid architecture and summary of binding interfaces of capsid with diverse host factors. During the maturation of HIV-1 virion, ~1500 CA monomers assemble into ~250 hexamers and exactly 12 pentamers in alignment produce fullerene cone geometry, forming capsid (Figure 2A–D). The interfaces within the capsid which bind diverse host factors are highlighted in different colors, as detailed in the Figure 2E legend. (**A**) Structure of the CA monomer (PDB ID: 4XZF). The left structure is shown in surface representation and the right structure as ribbon representation. CA consists of two α-helical domains, an N-terminal domain (NTD) labeled in gold and a C-terminal domain (CTD) labeled in light blue, connected by a flexible linker (grey). All α-helices (H1-H11) and key structural elements are indicated by arrows with names. (**B**) Structure of a CA hexamer with top view (left) and side view (right) (PDB ID: 4XZF). (**C**). Structure of high-order (hexagonal) CA hexamers with top view (left) and side view (right) (PDB ID: 4XZF). (**D**). Architecture of the capsid (PDB ID: 3J3Y). The incorporation of pentamers (orange) at either end of capsid provides the curvature necessary to close the conical structure. (**E**) The interfaces within capsid that can bind diverse host factors are highlighted in different colors, which are shown in Figure 2A–D. Host proteins CypA, Nup358, and TRN-1 bind to the flexible CypA-binding loop (labeled in red) exposed on the top of CA-NTD that protrudes from the capsid outer surface. Host proteins CPSF6 and Nup153 share the same phenylalanine-glycine binding pocket (labeled in green) created by the NTD-CTD interface between two neighboring CA monomers in a hexamer. The restriction factor Mx2 specifically binds to the three-fold inter-hexamer interface (labeled in purple) of capsid; moreover, it is unable to bind to a CA monomer or a single hexamer. Host protein FEZ1 and metabolite IP6 can bind to the positively charged central pore of hexamers formed by R18 (labeled in dark blue) of CA. TRIM5α can form a hexagonal network (labeled in pink, shown in Figure 2D, also called TRIM5α cage) that avidly binds the capsid shell. The extent to which the TRIM5α cage can cover the capsid and how TRIM5α directly contacts the capsid surface have not been fully established.

The viral capsid shell consists of roughly 1200–1500 monomeric capsid proteins (named CA) assembled into approximately 250 CA hexamers and exactly 12 CA pentamers producing fullerene cone geometry [7,32,33] (Figure 2A–D). Five and seven CA pentamers incorporated at the narrow and broad end of the cone respectively, close the capsid and induce the characteristic curvature. This cone-like capsid measures ~60 nm at its broad end and tapers to 20–30 nm (Figure 2D). However, capsid shapes can vary from the cone-like geometry to tube-like structures with capped ends [34]. As an α-helical protein, each CA has two structurally distinct domains, an N-terminal domain (CA-NTD) and a C-terminal domain (CA-CTD), connected by a flexible linker (Figure 2A). The CA-NTD contains seven α-helices (α1–α7), a characteristic extended cyclophilin A (CypA)-binding loop and a β-hairpin, whereas CA-CTD consists of four α-helices (α8–α11), a short 3_10_-helix, and the major homology region (MHR). The assembly and stability of the capsid shell is driven by three sets of intermolecular protein–protein interactions between CA subunits: (1) intra-hexameric NTD-NTD contacts between individual CA molecules stabilize the hexamers and pentamers that function as the building blocks of the capsid, as well as form a central pore gated by the β-hairpin, (2) intra-hexameric NTD-CTD contacts between adjacent CA molecules further stabilize the individual hexamers and pentamers, and (3) inter-hexameric CTD-CTD contacts participate in dimeric and trimeric interactions to link the individual hexamers and pentamers [34,35,36] (Figure 2D). Therefore, the capsid core not only shields the viral genome but also provides expansive and diverse interfaces for cellular protein interactions. The unique capsid architecture underlies intrinsic core stability and provides for capsid–host factor interactions.

### 2.2. Capsid-Host Factor Interactions Contribute to Trafficking and Capsid Stability in the Cytoplasm

The host cytoplasm is a complex and molecularly crowded environment in which the HIV-1 capsid is likely too large to freely diffuse to the nucleus. Studies show that HIV-1 capsid traffics towards the nucleus along the microtubule (MT) network by hijacking adaptor and motor proteins [9]. MT network trafficking is achieved through dynein and kinesin motors and is a primary means of intracellular transport in cells [37]. Dyneins facilitate inward movements to the nucleus, and in contrast, kinesins mediate movements toward the cellular periphery. Bicaudal D2 (BICD2) is a dynein adaptor protein that was found to interact with capsid directly and links it to the dynein motor complex, facilitating its inward cytoplasmic trafficking [38,39]. Depletion of BICD2 had no effect on reverse transcription but resulted in a significant reduction of capsid nuclear entry. Interestingly, Fasciculation and Elongation Protein Zeta 1 (FEZ1), a kinesin-1 adaptor protein that usually transports cargo towards the periphery of the cytoplasm, was identified to link capsid to kinesin for transport by binding to the central pore within the CA hexamer with high affinity (Figure 2) [40,41]. However, why HIV-1 capsid has evolved to employ opposing motors to mediate inward trafficking, producing a potential bi-directional “tug-of-war” (Figure 1), and how BICD2 and FEZ1 function in concert to achieve that, remain to be elucidated. Furthermore, a yeast two-hybrid screen identified two microtubule-associated proteins, MAP1A and MAP1S, as interaction partners for CA [42]. It was proposed that MAP1 proteins contribute to inward trafficking by tethering capsids to the MTs. These findings suggest a complex interaction of capsid with the host cytoskeleton network, which guarantees the directed movement of the capsid towards the nucleus.

Cyclophilin A (CypA) is a peptidyl-prolyl *cis*-*trans* isomerase (PPIase), which binds to the flexible CypA-binding loop exposed on the top of CA N-terminal domain that protrudes from the capsid outer surface [43] (Figure 2). A recent study showed that CypA is also able to recognize specific geometries of the curved conical capsid, simultaneously interacting with three CA protomers from adjacent hexamers via two noncanonical interfaces [44]. CypA–capsid interactions can modulate the capsid stability in a cell type-dependent manner during its cytoplasmic trafficking until capsid arrives and docks at the cytoplasmic side of the nuclear pore to initiate nuclear entry [16,45,46,47].

In addition to cellular proteins that interact with capsid in the cytoplasm, the cellular metabolite inositol hexaphosphate (IP6) also exerts a physical interaction with capsid during infection. IP6 is a negatively charged small molecule that binds to the positively charged central pore of the CA hexamer (Figure 2), thereby promoting capsid stability [48,49]. The hexamer central pore also acts as a channel for the transport of deoxynucleotide triphosphates (dNTPs) into capsid, offering substrates for reverse transcription that occurs inside the core [50]. Some studies suggest that IP6 binding governs the transportation of dNTPs [48,49,51], and others indicate that IP6 loss may drive rapid uncoating while capsid is traversing through the nuclear pore [52,53]. Many questions on IP6 function still remain to be answered and the capsid–metabolite interactions deserve further studies.

### 2.3. Roles of Capsid in Nuclear Import

The interaction between capsid and NPC has drawn attention given that, unlike other retroviruses which require cell division and the breakdown of the nuclear envelope to access the host genome, HIV-1 can infect non-dividing cells such as primary macrophages by hijacking host nucleoporins (Nups) for nuclear import [54,55]. However, capsid measures ~60 nm at its broad end and exceeds the inner diameter of NPC channel which is only ~40 nm [56]. Based on this discrepancy in size, it has been argued that capsid uncoating has to occur—at least partially—prior to nuclear import [57]. Two models for nuclear import of the viral genome have been proposed. In one model, capsid undergoes uncoating during cytoplasmic trafficking in coordination with reverse transcription, the viral DNA and components of capsid lattice are then transported to the nuclear pore and after further remodeling, some form of a pre-integration complex (PIC) translocates to the nucleus [58,59,60]. The second proposed model is that the capsid remains intact until it docks at the NPC and initiates uncoating, followed by the translocation of PIC [61]. However, recent studies suggest a third model where capsid passes through NPC in an intact or nearly intact status, then completes reverse transcription inside the nucleus, followed by uncoating near the host cell genomic integration site [10,11,62,63,64]. A recent report using atomic force microscopy to evaluate Nup documents the variability of nuclear pore sizes, which suggests that nuclear pores may be of sufficient size to allow capsid to translocate through the NPC [10,65]. If these findings are substantiated, the roles of cytoplasmic host factors that interact with capsid will need to be reevaluated as to their functions in nuclear membrane recognition, NPC transport, and uncoating.

The NPC is a macromolecular structure assembled from ~30 different Nups, which is embedded in the double membrane of the nuclear envelope and controls both passive (small molecules) and highly regulated active transport (cargoes larger than ~40 KDa) [56,66,67,68]. Given the size of capsid, the NPC is considered as the only channel for capsid translocation into the nucleus, and apparently a rate-limiting step as well, since only a small fraction of capsid is successfully transported. Various genome-wide RNA interference (RNAi) screens identified a series of NPC components as essential host factors for HIV-1 infection (defined as HIV-1 dependency factors), such as Nup50, Nup62, Nup85, Nup98, Nup107, Nup133, Nup153, Nup155, Nup160, Nup214, and Nup358 [69,70,71,72]. The subsequent biochemical analysis highlighted that Nup358 and Nup153 are able to bind to capsid directly and mediate its nuclear import [73,74]. Nup358 is located on the cytoplasmic side of the NPC while Nup153 is located on the nucleoplasmic side (Figure 1).

Evidence suggests that capsid may dock at the NPC via interaction with Nup358, a filamentous protein containing glycosylated phenylalanine–glycine (FG) repeats [75]. Nup358 has a CypA homologous domain (CHD) which can bind to the CypA-binding loop exposed on capsid surface [73,76] (Figure 2). Since Nup358 and CypA share the same binding loop, it has been suggested that Nup358 may act as a docking station and binds the capsid presented from CypA, assisting capsid to recognize the NPC channel entrance [54,73,77]. Another possibility is that capsid docks to the NPC with the pentamer-rich end, preferably the narrow end. Pentamers of the cone represent specialized binding sites that are recognized by cyclophilin domains contained in CypA and Nup358 [77], therefore, the stronger interaction of the narrow end with Nup358 guides the orientation of a capsid and facilitates the subsequent nuclear import. A number of studies also suggested that the peptidyl-propyl isomerase activity exhibited by Nup358 could destabilize the capsid core by causing cis-to-trans isomerization and therefore facilitates uncoating [75,78]. In addition, Transportin-1 (TRN-1), a nuclear transport receptor mediating nuclear import, also binds to capsid by engaging with the CypA-binding loop and may trigger uncoating during viral nuclear import [79]. This remarkable utilization of common capsid interfaces for interacting with diverse host factors indicates a highly dynamic capsid–host protein interplay. However, how these interactions are regulated in a spatiotemporal fashion to assist nuclear entry still remains largely unknown.

Upon entry into the NPC, Nup62, which is localized on the central channel, has been postulated to interact with capsid. This is based on biochemical and in vitro evidence that Nup62 interacts with CA tubes. However, knock-down of Nup62 had little effect on HIV-1 infectivity [80]. A recent cryo-electron tomography (cryo-ET) report has localized and visualized capsids in NPCs [10]. It yet remains to be determined how capsid crosses the central channel of NPCs and what the roles of Nups are in this process. The inducible NPC blockade system may prove useful to evaluate capsid nuclear import by trapping capsids as they transverse NPCs [11,81]. Because of the rarity and dynamic features of productive infection, this methodology may allow capsid and NPC visualization with cryo-ET.

As the capsid transverses the NPC, it must exit via the nuclear basket to the interior of the nucleus. Nup153 is a nucleoplasmically oriented Nup as well as a main member of the basket [56]. Its C-terminal contains 29 FG motifs, which are natively unfolded with no appreciable secondary structure. The FG motif at positions 1415–1418 binds to the NTD-CTD interface between two CA subunits of the hexamer [29,74,82,83] (Figure 2). Notably, Nup153 binds CA hexamers with much higher affinity than CA monomers [83], indicating that some hexameric CA still remains intact in the nuclear basket. Cleavage and polyadenylation specificity factor subunit 6 (CPSF6), which functions as a component of the cleavage factor I mammalian (CFIm) complex to determine mRNA polyadenylation sites, is another well-characterized host cellular protein interacting with capsid. The central proline-rich domain (PRD) (residues 314–322) of CPSF6 also contains a FG motif that binds directly to capsid in the NTC-CTD interface between two neighboring CA monomers in a hexamer (Figure 2), more specially, a hydrophobic pocket created by CA NTD helices 3, 4, 7, and by CTD helices 8 amd 9 from an adjacent monomer [82,83,84]. Strikingly, the capsid uses the same FG-binding pocket for the interactions of Nup153 and CPSF6, although there are differences that are specific for each ligand [82,83]. The capsid–CPSF6 interaction that occurs at the nuclear envelope was shown to contribute to the nuclear entry of capsid [85,86]. Other studies suggest, however, that the capsid–CPSF6 interaction, which happens in the nucleus, facilitates the release of PIC from the confines of the NPC by competitive binding with Nup153 to capsid and directs viral DNA integration to actively transcribed euchromatin [87,88]. However, more mechanistic insight is needed to address capsid entry through NPCs, such as how the interactions of capsid with Nup153 and CPSF6 are coordinated during nuclear import. Also important to understand is what triggers capsid uncoating and release of PICs as the capsid moves through the NPC. Does the process of moving through the NPC trigger uncoating? Of note, there is another opinion suggesting that the volume of rigid viral double-stranded DNA converted from relatively flexible single-stranded RNA cannot be accommodated inside the intact capsid and the resulting pressure might mechanically trigger uncoating [64,89].

### 2.4. Capsid-Targeting Host Restriction Factors

HIV-1 has evolved to take full advantage of cellular host factors for their lifecycle. As to be expected in the host-pathogen arms race, host cells have evolved as well to recognize and neutralize the activity of lentiviruses [90]. Cellular infection results in innate immunity responses, including production of IFNs and antiviral activities of the restriction factors [91,92]. When released into the cytoplasm, capsid can be directly targeted by at least 2 host restriction factors, the tripartite motif-containing protein 5 alpha (TRIM5α) and myxovirus resistance 2 (Mx2), although each disrupts infection via different mechanisms [14,93,94,95,96].

TRIM5α was initially characterized as a restriction factor for preventing cross-species (zoonotic) infection of HIV-1, yet not effective in controlling infection in the natural host [97]. It was found that TRIM5α can bind to capsid and causes premature uncoating of the core, resulting in reverse transcription inhibition, which is ultimately targeted to proteasomal degradation [93,98,99,100]. As to structure, TRIM5α contains a RING domain, a B-box domain, a coil domain, and a SPRY domain [99]. TRIM5α molecules can dimerize through coil domains and further oligomerize through the B-box domain to form a hexagonal network (Figure 2D), while utilizing the SPRY domain to bind to a large surface area of capsid, including the center of a single hexamer, between two adjacent hexamers and between three hexamers [101,102]. This binding mode significantly strengthens the interaction through avidity. The RING domain of the TRIM5α is responsible for the restriction function due to its ligase activity [100]. Interestingly, studies also found that HIV-1 can exploit capsid-binding proteins to mitigate host innate immunity. For instance, CypA bound on capsid can not only evade the recognition by immune sensor cGAS (cyclic GMP-AMP), but also block TRIM5α binding to the viral core [103,104].

Mx2 is a dynamin-like GTPase, although the GTPase activity is not necessary for anti-HIV-1 activity [105]. After being induced by IFNα, Mx2 is recruited to the cytoplasmic face of the nuclear envelope, the triple-arginine motif localized in N-terminal of Mx2 can specifically bind to the three-fold inter-hexamer interface of capsid (Figure 2) and block the nuclear import [95,106,107,108] (Figure 1). Of note, Mx2 is unable to bind to a CA monomer or single hexamer, demonstrating the critical role of intermolecular interfaces on capsid for interacting with host proteins [19].

Taken together, the many capsid–host interactions suggest that HIV-1 has evolved to take full advantage of cellular host factors via capsid expansive protein-docking outer surfaces. However, the spatiotemporal interactions of capsid with diverse host factors remains largely unknown. In light of the recent cryo-ET visualization of capsid in NPCs and nucleus, it begs the question as to roles of a diverse set of capsid-binding host factors in nuclear import and uncoating. Of note, mechanistic studies have been hampered by the fact that only a minority of the incoming capsids can successfully arrive at the nuclear envelope, be imported into the nucleus, and lead to eventual productive infection [61]. Better mechanistic and structural understanding of capsid–host interactions will undoubtedly provide insights on post-entry viral events and contribute to the development of capsid-centric antiretroviral therapies.

## 3. Recent Advances in HIV-1 Capsid Inhibitors

The role of capsid in the early stages of the HIV-1 replication cycle highlights capsid as an attractive antiviral target for inhibitors. The inhibition of either capsid assembly or capsid disassembly will be able to arrest HIV-1 replication and thus infection. Several promising capsid inhibitors have been reported, and among them, PF74 is the most studied [26]. The recently reported GS-CA compounds (GS-CA1 and GS-6207), which contain similar functional scaffolds with PF74, show greater potency and long-acting potential [28,29,109]. All three inhibitors bind to the same pocket occupied by the host proteins CPSF6 and Nup153 within the NTD-CTD inter-subunit interface, and thus promote capsid stability and interfere with capsid nuclear entry [83,110].

PF74 was first identified by Pfizer during a screen for inhibitors of HIV-1 replication [111]. PF74 halts HIV-1 replication at nanomolar levels (half-maximal effective concentration EC_50_ = 8–640 nM) by stabilizing capsid at early stages of replication or by distorting capsid lattice in the later stages of replication [111,112]. However, PF74 exhibits extremely poor metabolic stability [113], which limits its utility in clinic. In 2017, Gilead Sciences reported GS-CA1 as a next-generation capsid inhibitor, which inhibits HIV-1 replication in T-lymphocytes and peripheral blood mononuclear cells (PBMCs) with EC_50_ of 240 and 140 pM, respectively [114]. Two years later, Gilead Sciences rolled out GS-6207, a derivative of GS-CA1 which has an EC_50_ = 50–100 pM depending on the viral isolate [115]. Further studies showed that both GS-CA1 and GS-6207 block multiple steps during HIV-1 replication and show long-acting potential as inhibitors [110,116]. Given the excellent potential as a potent, long-acting antiretroviral compound, GS-6207 has entered clinical trials, and phase I clinical trials (NCT03739866) were recently completed showing a 6-month dosing interval and is now in phase II clinical trials (NCT04143594/NCT04150068) [110,117,118].

**Figure 3:**
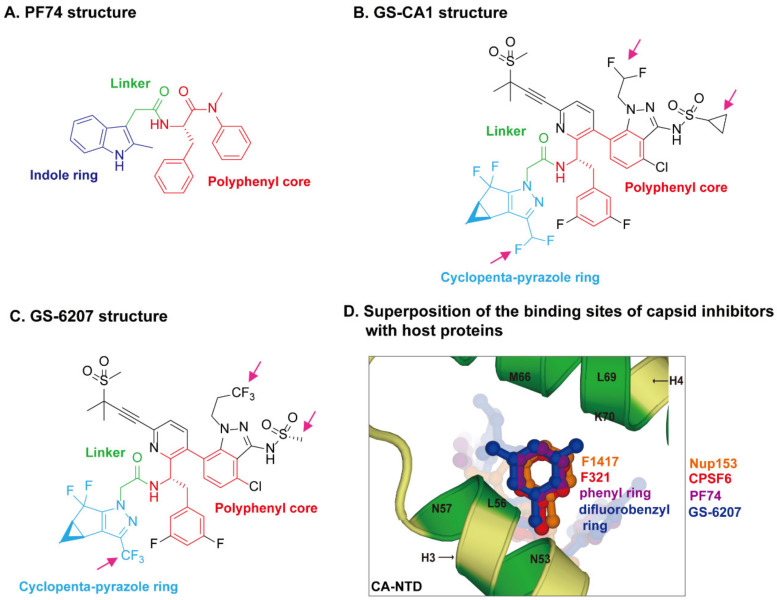
HIV-1 capsid inhibitors and the comparison of their binding sites on capsid with host proteins. (**A**) Chemical structure of PF74. PF74 contains a polyphenyl core (red), a linker region (green), and an indole ring (dark blue). (**B**) Chemical structure of GS-CA1. (**C**) Chemical structure of GS-6207. Gilead GS-CA compounds possess a similar polyphenyl core (red) and linker region (green) with PF74, and a cyclopenta-pyrazole ring (light blue), as well as other chemical groups (black). GS-6207 differs from GS-CA1 by three modifications (magenta arrows), difluoroethyl groups on indazole ring are replaced by a trifluoroethyl group, a cyclopropane moiety on sulfonamide group is replaced by a methyl group, and a difluoromethyl group on cyclopenta-pyrazole ring is replaced by a trifluoromethyl moiety. (**D**) Comparison of the binding sites of capsid inhibitors with host proteins. The capsid inhibitors GS-6207 and PF74 bind to the same pocket occupied by host proteins CPSF6 and Nup153 within the NTD-CTD inter-subunit interface created by two adjacent CA monomers in a hexamer. The structural comparison shows that the difluorobenzyl ring (dark blue) of GS-6207 and the phenyl ring (purple) of PF74 superpose well on the F321 (red) of CPSF6 and F1417 (orange) of Nup153.

The crystal structures of capsid bound to the peptides from CPSF6 (313–327) and Nup153 (1407–1423) have been resolved [83]. The crystal structures of PF74-bound CA hexamer show that PF74 binds in the same pocket with CPSF6 and Nup153 [82,83,119]. More specifically, the structural comparison revealed that F321 of CPSF6 and F1417 of Nup153 perfectly superpose on the phenyl ring of PF74 [120] (Figure 3D), which explains how PF74 interferes with CPSF6 or Nup153 binding to capsid and inhibits HIV-1 replication. The binding models of GS-CA compounds with capsid have recently been elucidated by computational docking approaches [120] and structural studies [29,110]. PF74 contains a polyphenyl core, a linker region, and an indole ring (Figure 3A). GS-6207 and GS-CA1 possess a similar polyphenyl core and linker region as PF74, and a cyclopenta-pyrazole ring (Figure 3B,C). One molecular docking study of the structure of GS-CA1/PF74 with CA hexamer showed that the shared polyphenyl cores of the two molecules superpose well, whereas the indole ring of PF74 superposes closely on the cyclopenta-pyrazole ring of GS-CA1 [120]. Besides, GS-CA1 has additional chemical groups such as the methanesulfonyl moieties, which are found to interact with the NTD of adjacent CA monomers. It is believed that these additional interactions lead to a higher binding affinity of CA hexamer with GS-CA1 than PF74, resulting in enhanced antiretroviral potency. Since GS-6207 differs from GS-CA1 by several modifications (Figure 3B,C), the researchers also docked GS-6207 in the crystal structure and found that GS-6207 binds to the same binding pocket as GS-CA1, but with a slightly better Glide score, indicating a better binding affinity. A recent study elucidated the structural and mechanistic bases as to how GS-6207 stabilizes viral core and thereby inhibits functional disassembly of the capsid shell in infected cells [29]. In that study, the X-ray structure of GS-6207 bound to a pre-stabilized CA hexamer revealed that GS-6207 binds in the hydrophobic pocket formed by two adjacent CA subunits with a stoichiometry of six GS-6207 compounds bound per each CA hexamer. There are extensive van der Waals and hydrogen-bonding interactions between GS-6207 and the hydrophobic pocket, with the binding of six inhibitors resulting in a more stable hexamer. The cryo-electron microscopy and hydrogen–deuterium exchange experiments further found that GS-6207 is able to promote distal intra- and inter-hexamer interactions that stabilize the curved capsid lattice. The potential of GS-6207 to stabilize the capsid core highlights the ability of the compound to halt capsid disassembly in infected cells. In addition, comparing GS-6207 binding to known interactions of CPSF6 and Nup153 with CA hexamers demonstrated substantial overlap between GS-6207 and the main chain of CPSF6, with F321 of CPSF6 superimposing on the di-fluorobenzyl moiety of GS-6207. Similarly, the backbone of Nup153 aligns along the polyphenyl core of GS-6207, with F1417 of Nup153 closely superimposing on the di-fluorobenzyl moiety. Therefore, as might be expected, GS-6207 interferes with capsid binding to the cellular host factors CPSF6 and Nup153. The detailed structural insights into the potent antiviral activity of GS-6207 will undoubtedly contribute to the rational development of second-generation therapies.

Collectively, these findings strongly imply that compounds targeting structural features that interfere with interactions between host proteins and capsid have the potential to be highly effective inhibitors of HIV-1 infection.

## 4. High-Throughput Screening Techniques Used to Reveal HIV-1–Host Interactions

HIV-1 has a relatively small genome, with many of the essential viral lifecycle functions accomplished through hijacking of host proteins. Given that HIV-1 proteins are able to interact with diverse cellular proteins, this leverages viral functions at the expense of the host during the progression of infection. To glean insights into the viral lifecycle and host factor contributions, as well as identify novel antiretroviral targets, a full understanding of the HIV-1–host cell “interactome” is necessary. The rapid advances in high-throughput genomic and proteomic screening approaches have provided an unbiased characterization of HIV–host interactions. In this section, we will briefly review and highlight functional genomic and proteomic approaches which have been successfully implemented in HIV-1 or other viral studies, their limitations, and the potential they hold for identifying capsid–host cell interacting proteins.

### 4.1. Genomic Approaches

Genome-wide screening technologies have offered unprecedented opportunities for interrogating host genes essential for HIV-1 replication. In 2008, three independent screens utilized small-interfering RNA (siRNA) to knock down >20,000 human genes in 293T or HeLa-derived cells, then evaluated alterations in HIV-1 infection and replication [69,70,71]. Each of the screens identified approximately 300 genes, but the overlap between any pair of screens only ranged from 3% to 6% [121]. The variation may be ascribed to the cells used for screening, experimental differences, timing of sampling, and/or different filtering criteria. Even so, these three siRNA screens identified 842 genes affecting HIV-1 replication when knocked-down, such as CD4, coreceptor CXCR4, the capsid interacting proteins CypA, Nup153, and Nup358, as well as the viral budding factor TSG101 [121]. Gene Ontology enrichment analyses from the siRNA screens identified host factors participating in cellular processes, with the highly ranked “Nuclear Pore/Transport” genes (21–24 genes in each screen), correctly implicating a central role for the NPC in HIV-1 nuclear import in Reference [121]. Later, CRISPR/Cas9 technology, which is short for clustered regularly interspaced short palindromic repeats and CRISPR-associated protein 9, a genome-wide knockout screening method with higher sensitivity and specificity, was applied to the study and further revealed additional host proteins linked to HIV-1 infection [122,123]. Although both methods are rapid and high-throughput in application and identify proteins and possible pathways, a limitation is that viral-host factors identified by these approaches may act only indirectly. To identify a physical interaction with HIV-1 proteins, further characterization requires biochemical or other types of protein-based assays. Additional limitations are that host factors whose disruption or depletion are cytotoxic or lethal to the cells can be lost in genetic screens. Table 1 provides a summary of the various approaches for identifying HIV-1–host factor interactions and their advantages and limitations.

### 4.2. Proteomic Approaches

The application of proteomic techniques to virology have facilitated significant insights of viral-host protein interactions. A widely used technique has been yeast two-hybrid (Y2H) screening and Affinity Purification followed by Mass Spectrometry (AP-MS) to identify isolated proteins. Recently, protein–protein proximity-dependent labeling approaches have been used to identify closely localized viral-host protein interactions. Briefly, these approaches utilize a viral protein of interest (bait) for identifying potential interacting host (prey) proteins.

### 4.3. Yeast Two-Hybrid (Y2H) Screen

**Figure 4:**
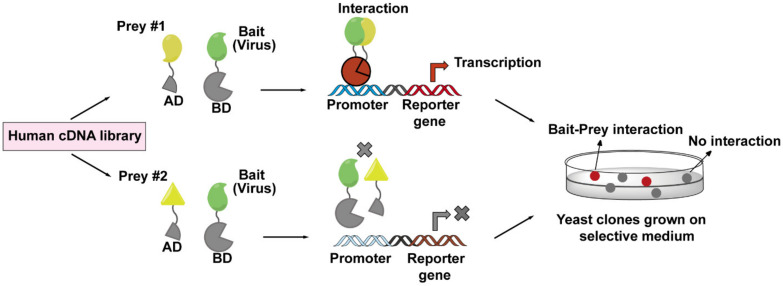
The workflow of yeast two-hybrid (Y2H) screen employed to identify virus–host interactions. The principle of a Y2H system is based on the reconstitution of a functional transcription factor driven by the interaction between a bait protein and a prey protein. The DNA-binding domain (DB) of the transcription factor is fused to a viral protein of interest (bait, in green), while the transcription activation domain (AD) of the transcription factor is fused to a host protein (prey, in yellow) coming from human cDNA libraries. Upon co-expression of the bait and prey fusions in yeast cells, if the bait and prey interact, DB and AD will be reconstituted (indicated in red), and thus activate the transcription of a reporter gene (top lane), whose expression can cause a visible color change on the selective plate. These positive clones can be isolated for sequencing to determine the prey proteins. If the bait and prey do not interact, DB and AD will remain separated, and transcription of the reporter gene does not occur (bottom lane).

A Y2H screen is based on the transcription of a reporter gene driven by the interaction between a viral bait protein and a host prey protein. The cartoon (Figure 4) diagram shows a viral protein acting as a bait protein and is fused to the DNA-binding domain (DB) of a transcription factor. The prey protein is fused to the transcription activation domain (AD). Usually, the prey proteins are from human cDNA libraries that may represent all possible proteins expressed in various HIV-1 target cells. Both protein chimeras are then expressed in yeast cells and, if binding occurs between bait and prey protein, an active transcription factor is reconstituted, leading to the expression of the reporter gene. With this method, the interactions between baits (viral protein) and preys (host proteins) can be easily monitored and detected. A limitation, however, is that Y2H screens are performed in yeast cells rather than viral-infected host cells, which may result in artificial interactions due to the forced colocalizations in the nucleus or loss of signal due to the misfolding of the bait and prey proteins [124]. Therefore, the addition of an in situ confirmation step through a second method is always necessary for the verifying virus–host interactions discovered by Y2H screens. The host factors MAP1A and MAP1S that facilitate the cytoplasmic trafficking of HIV-1 capsid were found as interaction partners for CA by this method [42].

### 4.4. AP-MS Combined with Stable Isotope Labeling with Amino Acids in Cell Culture (SILAC) or Chemical Cross-Linking

**Figure 5:**
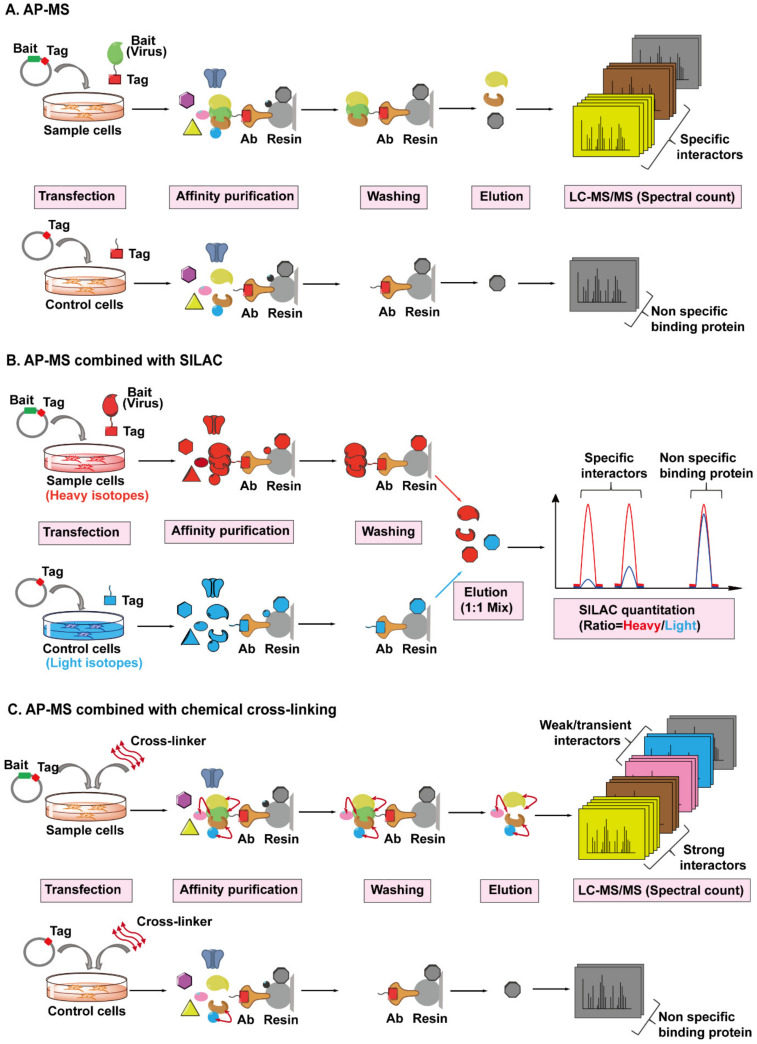
The workflows for AP-MS combined with SILAC or chemical cross-linking employed to identify virus–host interactions. (**A**) The workflow of AP-MS. As shown in the top lane, a typical AP-MS experiment begins with the fusion of an affinity tag (red) into a viral protein of interest (bait, in green). The tagged bait protein is then introduced into the host cells by transfection. After a period of expression, the tag-targeting antibody (bronze) conjugated to a resin (light grey) is used to pull down the bait protein, along with any host proteins (preys) bound to it. The resin should be washed several times to remove non-specific binding proteins (black). The captured prey host proteins (yellow, brown, and grey) are subsequently eluted from the resin, digested, and analyzed by Liquid Chromatography-Tandem Mass Spectrometry (LC-MS/MS). In parallel, a control experiment is set up by only introducing the affinity tag into the host cells, followed by the exact same steps performed in the sample experiment, as shown in the bottom lane. By comparing the number of identified MS/MS spectra of the same protein from sample or control cells, the viral protein-specific interactors (yellow and brown) can be distinguished from the non-specific binding proteins (grey) attached to the resin. (**B**) The workflow of AP-MS combined with SILAC. Sample cells labeled with heavy isotopes (red) are transfected with tagged bait plasmid, whereas control cells labeled with light isotopes (blue) are transfected with tagged empty plasmid, followed by parallel affinity purification, washing, and elution. The eluted prey proteins from different host cells are mixed together, digested, then analyzed and quantified by LC-MS/MS. Each captured host protein owns a heavy/light ratio indicating its specificity of interaction with the bait protein. SILAC-based quantification is more accurate than spectral count-based quantification. (**C**) The workflow of AP-MS combined with chemical cross-linking. After the transfection and expression of tagged bait protein or tag in host cells, cross-linkers (red double ended arrows) are added into cells to preserve the weak and transient interactions (pink and blue) between bait and prey proteins, then followed by AP-MS, as detailed above.

AP-MS has been the most common and adaptable method for studying virus–host protein interactomes [125,126]. A conventional AP-MS workflow (Figure 5A) usually begins with fusing an affinity tag into a viral bait protein of interest since antibodies toward viral proteins are often unavailable or expensive to purchase, then the viral bait protein is introduced into target cells by transient transfection or stable expression. After a period of expression (considered as a mimic of infection), an antibody conjugated to a resin is used to pull down the viral protein, along with any host proteins that are bound. The captured host prey proteins are subsequently eluted from the resin and analyzed by the MS. However, several technical challenges need to be taken into consideration when performing this workflow [127,128]. Firstly, AP screens are prone to false positive results due to non-specific binding of abundant and “sticky” proteins to the resin during the pull-down step, and to address this, a control sample is always set up in parallel. Additionally, the interaction between viral and host proteins has to be strong enough to survive the co-precipitation process and weak or transient interacting proteins will be lost. Therefore, several methods have been introduced to solve these limitations. In order to reduce non-specific interactions, tandem affinity purification (TAP), a procedure using two affinity mechanisms sequentially to precipitate the virus–host protein complexes, has been widely implemented [129,130]. In a TAP experiment, two different tags are inserted into the viral bait protein, followed by subsequent purification of the bait protein in two sequential affinity steps, which can obtain a cleaner purification of prey proteins compared to one-step isolation.

Another attractive approach is to integrate quantitative proteomic strategies such as Stable Isotope Labeling with Amino acids in Cell culture (SILAC) into AP-MS workflow (Figure 5B). SILAC-based quantitative MS is a powerful tool to discriminate specific and non-specific interactions during pull-down assays [131,132]. Cells expressing the viral bait protein and parallel control cells are labeled with different amino acid isotopes (heavy and light), then both types of cells are mixed equally, followed by AP-MS, as detailed above. Each co-purified host protein bound to the viral protein can be quantified and each protein has a heavy/light ratio. A high ratio indicates a potential specific interaction while nonspecific binding leads to a ratio near 1.

However, these methods are not helpful for studying weak and transient interactions. An approach called cross-linking is introduced into AP-MS pipeline to stabilize weak interactions and preserves transient protein interactions in cells (Figure 5C). A chemical cross-linker, which contains at least two reactive groups that flank a linker region, is able to react with particular amino acid side chains localized in two adjacent proteins and covalently link them, thereby preserving weak and transient noncovalent protein interactions [133]. Additionally, the cross-linked amino acid residues at the interaction interphase can be identified by MS and provide surface topology information [134]. To date, the AP-MS approach, or coupled with SILAC or chemical cross-linking methods, have been successfully implemented to globally map HIV–host interactomes. For example, an ambitious investigation conducted in 2012 individually expressed all HIV-1 proteins fused with a strep or FLAG tag, then performed AP-MS separately [30]. They identified with high confidence 497 HIV-human protein–protein interactions, providing the first global landscape of HIV-human protein complexes. In another study investigating potential interaction partners of the HIV-1 structural protein Gag, researchers performed six independent AP-MS and identified 1804 candidates in total, while SILAC screen was used to distinguish specifically co-purifying interactors and highlight the strongest candidates [135].

### 4.5. Proximity-Dependent Labeling (PDL) Technology

**Figure 6:**
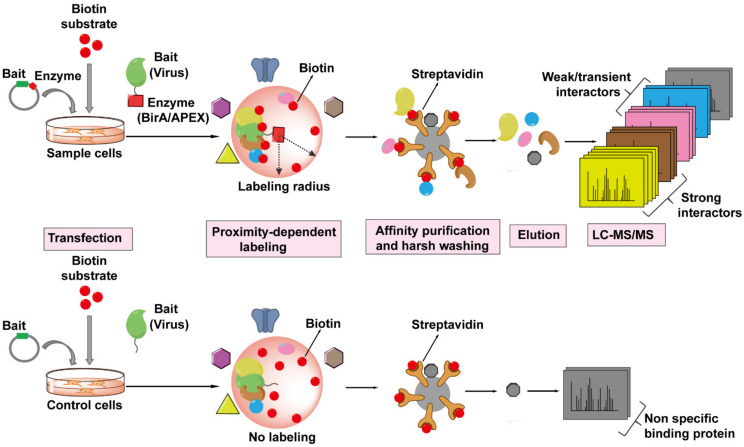
The workflow of Proximity-Dependent Labeling (PDL) technology followed by MS to identify virus–host interactions. The viral protein of interest (bait, in green) is fused to an engineered biotin ligase, BirA*, or ascorbate peroxidase enzyme, APEX (indicated by red square), and expressed in host cells, whereas only bait is expressed in the cells as control. The bait-enzyme when supplied with its appropriate substrates generates reactive intermediates which then covalently label all proximal host proteins (prey) within a radius of ~10–20 nm with biotin (indicated by red balls). The biotinylated proteins (prey) are isolated from host cells and enriched by streptavidin-conjugated beads, proteins are then eluted from the beads, digested, and then identified by LC-MS/MS. The PDL approach can label any host proteins that are within the labeling radius of the viral protein-enzyme, whether directly (strong, weak, or transient interactions) or not directly associated with the viral protein.

PDL technology is a newer generation of high-throughput screening techniques for probing protein–protein interactions. The basic principle of PDL requires the fusion expression of the viral (bait) protein of interest with an enzyme which can covalently label neighboring proteins (prey) in situ with biotin molecules in a distance-dependent manner in the presence of appropriate substrate (Figure 6). Biotinylated proteins are isolated from cells and precipitated by binding to streptavidin-coated matrix and eluted for MS analysis. This approach can capture any host proteins (prey) within a radius of ~10–20 nm of the viral protein-enzyme that are directly or not directly associated with the viral protein-enzyme. In addition, the binding affinity of biotin to streptavidin is one of the strongest non-covalent bonds and thus allows for extremely stringent washing conditions of the matrix to remove non-specific binding proteins. PDL can also be coupled with SILAC to further decrease the background noise. So far, there are two kinds of enzymes that can mediated the biotinylation of nearby proteins suitable for PDL.

One type of PDL uses the *E. coli* biotin ligase BirA* with a site mutation R11G (also termed BioID), which can tag a biotin molecule to the lysine of nearby protein within 10 nm range in the presence of biotin and ATP [136]. The bait protein can even target BirA* to specific subcellular locations, such as the nuclear envelope, allowing scientists to probe interactions in locations of interest. For example, one study successfully utilized this approach to probe the architecture and constituents of nuclear pore complex (NPC) by fusing BirA* to different Nups localized in NPC [137]. This technology should be amenable to probe the architecture of or host factors interacting with capsid during nuclear import. In addition, BirA* has been fused to the Gag protein to identify its binding partners in Jurkat cells and identified DDX17 and RPS6 as novel host factors [138].

The second type of PDL relies on the engineered ascorbate peroxidase APEX enzyme and its mutant APEX2. Both enzymes can catalyze the oxidation of biotin-phenol to the short-lived biotin-phenoxyl radical in the presence of hydrogen peroxide, which then reacts with electron-rich amino acids such as tyrosine on neighboring proteins, within a 20 nm radius, resulting in their biotinylation [139,140]. APEX2 can label neighboring proteins in minutes rather than hours found for the BirA*. However, APEX2 appears to be more toxic to living cells than BirA*. In 2018, the Ting Lab developed TurboID, having 15 mutations compared to wild-type BirA, to enable biotin labeling in 10 min without toxicity [141]. As of yet, APEX2-based or TurboID-based proximity labeling has not been used for HIV–host protein interactome studies.

Currently, there are no publications we are aware of utilizing capsid as bait to perform any form of AP-MS to identify capsid-interacting host proteins at the cellular proteome level. Whether this reflects the difficulty of applying newer proteomic technologies to identify capsid–host proteins or the lack of identifying new host factor proteins using available technologies is unknown. However, there are methodological difficulties to circumvent to move the identification of host proteins that interact with capsid forward. These difficulties include the observation that few capsids survive in the cytoplasm after viral fusion for transport to the nucleus for a productive infection [61]. This may be overcome by increasing the multiplicity of infection, which could allow sufficient cellular capsid for proteomic studies. Engineering capsid with affinity tags or PDL enzymes would provide a suitable means for identifying capsid interacting proteins. However, modified capsid must retain its appropriate architecture to produce infectious HIV-1 for cellular studies. Given the recent report which successfully engineered CA with GFP and produced infectious virus for nuclear entry studies [63], it may be possible to use a similar strategy for affinity tags or PDL enzymes. This could allow proximity-dependent labeling strategies to capture capsid interacting host proteins, which would be identified by LC-MS/MS.

The genomic and proteomic approaches described herein are both specific and highly complementary to each other (Table 1). The combinations and comparisons of datasets produced from multiple high-throughput screening approaches serve to combat the limitations of each technique, while significantly increasing the confidence of identified target proteins. To date, many of these omics findings on HIV–host interactomes have remained in the literature, while some of the findings have been included in six public databases that are focused on pathogen–host interactions [31,142,143,144,145,146,147]. The “NCBI HIV-1 human interaction database”, which was created specifically for HIV-1 research, contains two types of interactions: “replication interactions”, the human genes that have been reported to affect viral replication and infectivity after knock-down/out, and“protein interactions”, the human proteins that have been reported to interact with HIV-1 proteins directly or indirectly [142]. It is worth mentioning that a majority of the interactions recorded in the databases were gleaned from omic studies but have yet to be functionally validated. It will be necessary to confirm the HIV-1 role of the various cellular proteins of interest by additional orthogonal functional assays, such as biochemical, optical, and conventional molecular virology methods.

## 5. Perspective

Clearly, the capsid has evolved to favorably interact with a number of distinct host factors. Given the versatility of host factors for supporting viral replication, a number of open questions remain: How do different host factors interact with the capsid in a coordinated manner or in a spatiotemporal fashion to facilitate capsid cytoplasmic and NPC transport? Are there yet to be discovered host factors that participate in these processes? We believe that the omics revolution will accelerate the identification of novel host factors that interact with capsid. Structural information gleaned from novel capsid–host factor interactions will provide both mechanistic insights into HIV-1 pathogenesis and small-molecule targeting locations. GS-6207 (Lenacapavir) stabilizes capsid and competes with the capsid binding host factors CPSF6 and Nup153, thereby disrupting nuclear import [29]. These findings imply that compounds targeting structural features that interfere with interactions between host proteins and capsid have the potential to be highly effective inhibitors of HIV-1 infection.

## Figures and Tables

**Table 1:** Summary of high-throughput screening techniques.

	Approaches	Advantages	Limitations
Genomic approaches	RNAi	Reduces gene expression at the mRNA level (knockdown), suitable for studying essential genes. The knockdown effect is transient, can be used for temporary loss-of-function study. Rapidly interrogate gene function at the level of the genome.	May generate false negatives due to incomplete silencing of the gene or causes cell toxicity. Suffers from high off-target effects. Poor reproducibility between experiments. Temporary silencing requires a narrow assay window.
	CRISPR	Completely and permanently silences genes at the DNA level (knockout), resulting in a robust signal. Fewer off-target effects than RNAi. Rapidly interrogate gene function at the level of the genome.	Knockouts of essential genes are lethal to the cells.
Proteomic approaches	Yeast two-hybrid (Y2H)	Only a cDNA library or a specific gene of interest is needed, which costs less compared to classical biochemical approaches. Rapid isolation of interacting proteins.	The fused proteins may not fold correctly. The forced colocalization of baits and preys in the nucleus results in high rates of false positives and false negatives. Difficult to detect interactions caused by protein modification.
	AP-MS	Relative ease of set-up. Suitable for label-free quantification. Suitable for strong or stable protein interactions study.	Prone to non-specific binding proteins contamination during pull-down step. Weak and transient protein interactions will be lost during pull-down step. Difficult to detect low abundant proteins.
	AP-MS coupled with SILAC	Accurate quantification of small differences in relative protein abundance. Can discriminate specific and non-specific protein interactions during pull-down assays.	Expensive SILAC reagents. Time-consuming in sample preparation. Complexity of MS spectra.
	AP-MS coupled with chemical crossing-linking	Can capture strong, weak, and transient protein interactions. The identified cross-linked residues/peptides provide structural and topological information for protein interactions.	Cross-linkers have no access to the sites within protein complexes. May capture indirect interacting proteins within protein complexes.
	Proximity-dependent labeling (PDL)	Can capture strong, weak, and transient protein interactions. The association of biotin with streptavidin allows extremely stringent washing conditions to remove non-specific binding proteins, reducing background.	May capture non-interacting proteins in close proximity.

## Data Availability

Not applicable.

## Abbreviations

AIDS	acquired immune deficiency syndrome
AP-MS	affinity purification followed by mass spectrometry
ART	anti-retroviral therapy
BICD2	Bicaudal D2
CA	monomeric capsid protein
CFIm	cleavage factor I mammalian
CHD	CypA homologous domain
CPSF6	Cleavage and polyadenylation specificity factor subunit 6
CRISPR/Cas9	clustered regularly interspaced short palindromic repeats and CRISPR-associated protein 9
CypA	cyclophilin A
cyro-ET	cyro-electron tomography
DmrB	drug-inducible dimerization domain B
dNTPs	deoxynucleotide triphosphates
FDA	Food and Drug Administration
FEZ1	Fasciculation and Elongation Protein Zeta 1
HIV-1	The Human Immunodeficiency Virus type 1
IFNs	interferons
IN	integrase
IP6	inositol hexaphosphate
LC-MS/MS	liquid chromatography-tandem mass spectrometry
MHR	major homology region
MT	microtubule
MX2	myxovirus resistance 2
NPC	nuclear pore complex
Nup	nucleoporin
PDL	proximity-dependent labeling
PIC	pre-integration complex
PPIase	peptidyl-prolyl cis-trans isomerase
PPIs	protein–protein interactions
RING	Really Interesting New Gene
RNAi	RNA interference
RT	reverse transcriptase
SILAC	Stable Isotope Labeling with Amino acids in Cell culture
siRNA	small-interfering RNA
SPRY domain	SPla and the RYanodine Receptor
TAP	tandem affinity purification
TRIM5α	tripartite motif-containing protein 5 alpha
TRN-1	Transportin-1
Y2H	yeast two-hybrid
